# The luciferase-based *in vivo* protein–protein interaction assay revealed that CHK1 promotes PP2A and PME-1 interaction

**DOI:** 10.1016/j.jbc.2024.107277

**Published:** 2024-04-06

**Authors:** Sana Ando, Keiko Tanaka, Maharu Matsumoto, Yuki Oyama, Yuri Tomabechi, Atsushi Yamagata, Mikako Shirouzu, Reiko Nakagawa, Noriaki Okimoto, Makoto Taiji, Koichi Sato, Takashi Ohama

**Affiliations:** 1 Laboratory of Veterinary Pharmacology, Joint Faculty of Veterinary Medicine, Yamaguchi University, Yamaguchi, Japan; 2 Laboratory for Protein Functional and Structural Biology, RIKEN Center for Biosystems Dynamics Research, Yokohama, Kanagawa, Japan; 3 Laboratory for Cell-Free Protein Synthesis, RIKEN Center for Biosystems Dynamics Research, Kobe, Hyogo, Japan; 4 Laboratory for Computational Molecular Design, RIKEN Center for Biosystems Dynamics Research (BDR), Osaka, Japan; 5 Drug Discovery Molecular Simulation Platform Unit, RIKEN Center for Biosystems Dynamics Research (BDR), Osaka, Japan; 6 Research Institute for Cell Design Medical Science, Yamaguchi University, Yamaguchi, Japan

**Keywords:** CHK1, DNA damage, NanoBiT system, PME-1, PP2A

## Abstract

Protein phosphatase 2A (PP2A) is an essential serine/threonine protein phosphatase, and its dysfunction is involved in the onset of cancer and neurodegenerative disorders. PP2A functions as a trimeric holoenzyme whose composition is regulated by the methyl-esterification (methylation) of the PP2A catalytic subunit (PP2Ac). Protein phosphatase methylesterase-1 (PME-1) is the sole PP2Ac methylesterase, and the higher PME-1 expression is observed in various cancer and neurodegenerative diseases. Apart from serving as a methylesterase, PME-1 acts as a PP2A inhibitory protein, binding directly to PP2Ac and suppressing its activity. The intricate function of PME-1 hinders drug development by targeting the PME-1/PP2Ac axis. This study applied the NanoBiT system, a bioluminescence-based protein interaction assay, to elucidate the molecular mechanism that modulates unknown PME-1/PP2Ac protein–protein interaction (PPI). Compound screening identified that the CHK1 inhibitors inhibited PME-1/PP2Ac association without affecting PP2Ac methylation levels. CHK1 directly phosphorylates PP2Ac to promote PME-1 association. Phospho-mass spectrometry identified multiple phospho-sites on PP2Ac, including the Thr219, that affect PME-1 interaction. An anti-phospho-Thr219 PP2Ac antibody was generated and showed that CHK1 regulates the phosphorylation levels of this site in cells. On the contrary, *in vitro* phosphatase assay showed that CHK1 is the substrate of PP2A, and PME-1 hindered PP2A-mediated dephosphorylation of CHK1. Our data provides novel insights into the molecular mechanisms governing the PME-1/PP2Ac PPI and the triad relationship between PP2A, PME-1, and CHK1.

Protein phosphatase 2A (PP2A) is the essential serine (Ser)/threonine (Thr) phosphatase that modulates more than 50% of Ser/Thr protein phosphorylation in cells (1). PP2A is a heterotrimeric holoenzyme consisting of a catalytic C subunit (PP2Ac), a scaffolding A subunit, and a regulatory B subunit. The B subunit, involved in substrate specificity, is derived from four gene families: PPP2R2 (B55), PPP2R5 (B56), PPP2R3 (PR72), and PPP2R6 (Striatin). Dysfunction of PP2A holoenzyme is involved in the pathogenesis of various diseases, including cancer and neurodegenerative diseases, and, notably, the suppression of PP2A activity is essential for the oncogenesis of human cells (2, 3). Therefore, strategies to restore PP2A activity are garnering attention in cancer research (4, 5).

The C-terminal Leu309 of PP2Ac undergoes methyl-esterification (methylation) catalyzed by leucine carboxyl methyltransferase 1 (LCMT1) (6). PP2Ac methylation promotes the formation of a subset of the PP2A holoenzyme (7, 8, 9). Protein phosphatase methylesterase-1 (PME-1) hydrolyzes the methyl group of PP2Ac Leu309 (10). PME-1 is the only PP2Ac methylesterase currently known, and PP2Ac demethylation does not occur in lysates extracted from PME-1 knockout (KO) cells (7). Transcriptome analysis of PME-1 KO cells showed that PME-1 regulates a wide range of intercellular signaling, including inflammation and epithelial-mesenchymal transition (11). Higher PME-1 expression is observed in various cancer types and is associated with a worse prognosis of gliomas, liver cancer, and prostate cancer (12, 13, 14), indicating that dysfunction of PP2A holoenzyme due to elevated PME-1 contributes to cancer development and malignant transformation. Consistent with this idea, suppression of PME-1 expression exerts anti-cancer effects on various cancer cell lines (13, 14, 15, 16, 17). However, there are cases where the results of PME-1 knockdown (KD) are not reproduced by the PME-1 inhibitor ABL127 (18, 19). The involvement of another function of PME-1 is considered a possible cause for this discrepancy. In addition to its role as a methylesterase, PME-1 exerts a direct inhibitory effect on the PP2A by binding to the active site of PP2Ac and the PP2A trimer (20, 21). Therefore, the function of PME-1 as a PP2A inhibitory protein might be a target for anti-cancer strategies. Nevertheless, the validity of this hypothesis is subject to discussion given that ABL127 hinders the interaction between PME-1 and PP2Ac in both *in vitro* pull-down assays and immunoprecipitation (7, 22). Hence, gaining a profound understanding of the PP2A regulation by PME-1 is essential for realizing therapeutic strategies targeting the PME-1/PP2A axis.

The NanoBiT system is utilized to identify the molecular mechanism that modulates protein-protein interaction (PPI) (23). In the NanoBiT system, divided luciferases, called LargeBiT (LgBiT) and SmallBiT (SmBiT), are tagged to the N- or C- terminus of the proteins interested. When these proteins are associated, luciferase is reassembled and becomes active. Therefore, PPI is measured as luminescent intensities in living cells (*In vivo* NanoBiT assay). In this study, *in vivo* NanoBiT system was applied for the PME-1/PP2Ac PPI analysis and revealed that ABL127 inhibits PME-1/PP2Ac PPI in living cells. Moreover, compound screening identified that a CHK1 inhibitor blocks PME-1/PP2Ac PPI. CHK1 phosphorylates PP2Ac to promote PME-1 association. Our finding provides novel insights into the molecular mechanisms governing the PME-1/PP2Ac PPI and the triad relationship between PP2A, PME-1, and CHK1.

## Results

### Establishment of the NanoBiT system for PP2Ac and PME-1 interaction

The combinations of tag types and positions were explored to obtain the optimal luminescence for PP2Ac and PME-1 interaction. Since a tag to the C-terminus of PP2Ac blocks PP2Ac methylation, only the N-terminal tag PP2Ac was tested. The combination of the C-terminus LgBiT of PME-1 and the N-terminus SmBiT of PP2Ac showed the highest luminescent intensity (Fig. S1*A*). The PME-1 R369D mutant exhibited reduced binding affinity with PP2Ac (20). We generated a plasmid with LgBiT added to the C-terminus of PME-1 R369D and compared its luminescent intensity with that of wild-type (WT) PME-1-LgBiT. The luciferase intensity associated with the PPI between PP2Ac and PME-1 R369D was more than 70% lower than that of PME-1 WT (Fig. S1*B*). These results suggest that this combination of tag type and position is appropriate for analyzing the PPI of PME-1 and PP2Ac. Therefore, we utilized lentivirus vectors to generate A549 cells stably expressing PME-1-LgBiT and SmBiT-PP2Ac (A549 PME-1/PP2Ac) (Fig. S1*C*).

**Figure 1:**
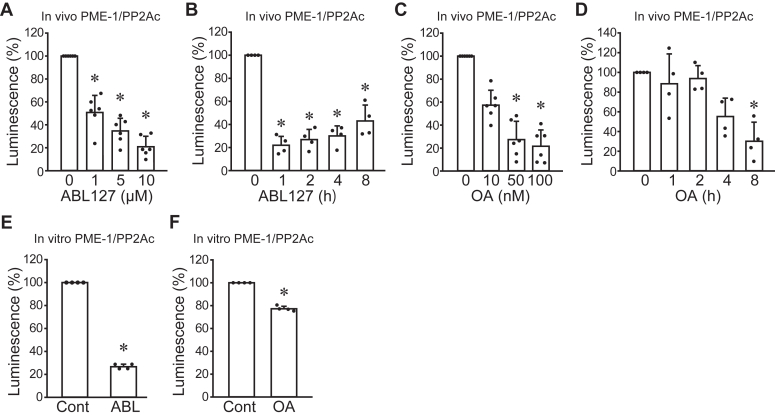
**Establishment of the NanoBiT system for PP2A and PME-1 interaction.***A*–*D*, A549 cells stably expressing LgBiT-PME-1 and SmBiT-PP2Ac (A549 PME-1/PP2Ac) were treated with ABL127 and okadaic acid (OA) to analyze concentration-dependent (*A*: ABL127, 24 h, *C*: OA, 8 h) and time-dependent (*B*: ABL127, 10 μM, *D*: OA, 100 nM) effects on luminescent intensity. *E* and *F*, *in vitro* NanoBiT assay was performed to analyze the effect of 1 μM of ABL127 (*E*) and 100 nM of OA (*F*) on PME-1/PP2Ac PPI luminescent intensity. ∗*p* < 0.05. Data points are independent biological replicates.

Our previous report showed that the PME-1 inhibitor ABL127 and the PP2A inhibitor okadaic acid (OA) reduced PPI between recombinant PME-1 and endogenous PP2Ac in a pull-down assay (7). Therefore, we analyzed whether these compounds inhibit the binding of PME-1 and PP2Ac in live cells using A549 PME-1/PP2Ac. Treatment with ABL127 did not affect the levels of SmBiT-PP2Ac and PME-1-LgBiT protein (Fig. S1, *D*–*F*). The ABL127 slightly, but not significantly, decreased PP2Ac protein, maybe due to the proteasomal degradation induced by hyper-methylation of PP2Ac (8). The luminescent intensity of A549 PME-1/PP2Ac cells rapidly decreased 1 h after treatment and showed a dose-dependent decline (Fig. 1, *A* and *B*). While OA did not affect PME-1-LgBiT protein levels, a 1.5-fold increase in SmBiT-PP2Ac protein was observed after OA treatment (Fig. S1, *G*–*I*), possibly due to a compensatory mechanism for inhibiting enzyme activity. Nevertheless, OA decreased the luminescent intensity of A549 PME-1/PP2Ac cells in dose- and time-dependent manners (Fig. 1, *C* and *D*). These data indicate that ABL127 and OA inhibited the PP2Ac and PME-1 association in live cells. To analyze the direct effects of these compounds on PME-1/PP2Ac association, SmBiT-PP2Ac, and PME-1-LgBiT were lysed from A549 PME-1/PP2Ac cells and utilized for *in vitro* NanoBiT assay. Both ABL127 and OA decreased PME-1/PP2Ac PPI luminescent intensity *in vitro* (Fig. 1, *E* and *F*), supporting the previous data that these compounds directly block PME-1/PP2Ac association.

To omit the possibility of the off-target effects on the luciferase, we generated LSmBiT: LgBiT and SmBiT were connected by a linker sequence. A549 cells stably expressing LgBiT (A549 LgBiT) were treated with ABL127 and OA. Although ABL127 reduced LSmBiT luminescence, the effect was less than 10% and considerably smaller than its impact on the PME-1/PP2Ac PPI luminescence (Fig. S1*J*). OA did not affect the luminescence of LSmBiT (Fig. S1*K*). *In vitro* NanoBiT assay using cell lysates from A549 LgBiT cells showed both ABL127 and OA did not decrease LSmBiT luminescent intensity (Fig. S1, *L*–*M*). These data demonstrate the utility of the NanoBiT system in analyzing the PME-1/PP2Ac PPI.

### CHK1 inhibitor suppresses the PME-1 and PP2Ac association

**Figure 2:**
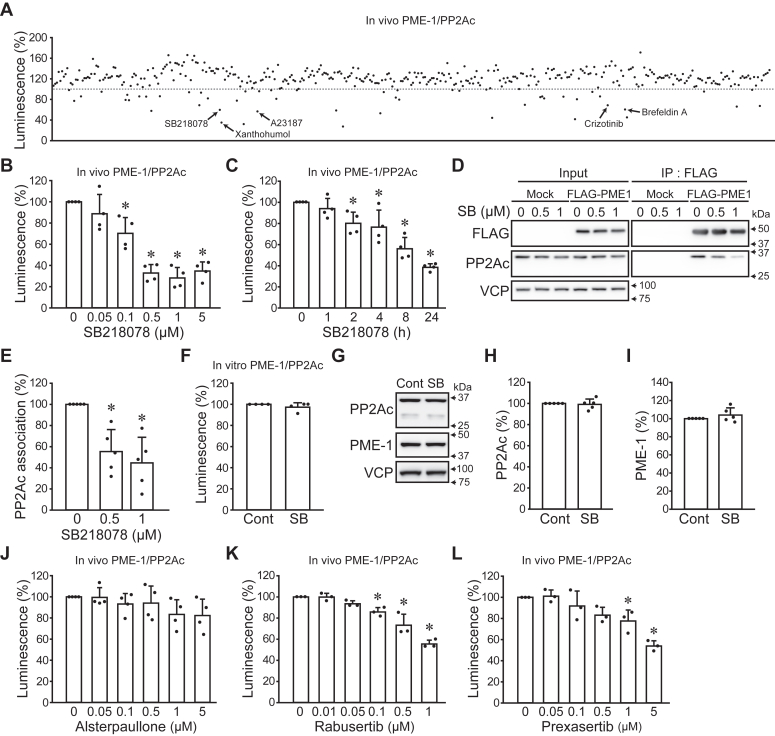
**CHK1 inhibitor suppresses the PP2Ac and PME-1 association.***A*, A549 PME-1/PP2Ac cells were treated with 399 compounds (5 μM) for 24 h, and luminescent intensity was analyzed. Data points were the means of the four samples from two independent experiments. *B* and *C*, A549 PME-1/PP2Ac cells were treated with SB218078, and the luminescent intensity was analyzed. The concentration- (*B*: 24 h) and time- (*C*: 1 μM) dependent effects were shown. *D* and *E*, shPME-1-resistant FLAG-PME-1 expressing vector or corresponding empty vector (mock) was transfected in A549 cells stably expressing shPME-1, followed by treatment with SB218078 (SB, 0.5, 1 μM) for 24 h. Immunoprecipitation was performed using FLAG-M2 beads. Representative images (*D*) and quantitative data (*E*) are shown. *F*, *in vitro* NanoBiT assay for the PME-1/PP2Ac PPI. SB218078 (1 μM) was applied to the cell lysate for 1 h. *G*–*I*, A549 cells were treated with SB218078 (1 μM) for 24 h and the protein levels were analyzed by immunoblotting. Representative images (*G*) and quantitative data (*H* and *I*) are shown. *J*–*L*, A549 PME-1/PP2Ac cells were treated with the indicated concentrations of alsterpaullone (*J*), rabusertib (*K*), and prexasertib (*L*) for 24 h and *in vivo* NanoBiT assay was performed. ∗*p* < 0.05. Data points are independent biological replicates.

Compound screening was performed using the *in vivo* NanoBiT system to identify the molecular mechanism for PME-1/PP2Ac PPI. A549 PME-1/PP2Ac cells were treated with 399 compounds (5 μM) for 24 h (Fig. 2*A*). This compound library includes various inhibitors and chemotherapeutic agents that are commercially available (Supplemental File 1). 17 compounds decreased the luminescent intensity of PME-1/PP2Ac PPI to less than 70%. The compounds that may be nonspecific, such as those that affect protein synthesis or degradation were excluded, and five compounds, SB218078 (CHK1 inhibitor), A23187 (Ca^2+^ ionophore), brefeldin A (Golgi inhibitor), crizotinib (EML4-ALK and ROS one inhibitor), and xanthohumol (DGAT inhibitor), were selected for the validation test. These compounds reduced the luminescence of the PME-1/PP2Ac PPI *in vivo* NanoBiT assay (Fig. S2*A*). On the other hand, with the exception of brefeldin A, all other compounds did not affect the luminescence of LSmBiT (Fig. S2*B*). CHK1 is a substrate of PP2A, and dephosphorylation of CHK1 by PP2A negatively regulates the cell cycle checkpoints (24, 25). Moreover, the public data of proteomics showed positive correlations of CHK1 with PP2Ac and PME-1, suggesting the functional relationship between CHK1 and PME-1/PP2Ac axis (Fig. S2, *C* and *D*). Based on these insights, we decided to focus on SB218078, a CHK1 inhibitor, for further investigation.

SB218078 decreased the luminescent intensity for PME-1/PP2Ac PPI in a concentration- and time-dependent manner (Fig. 2, *B* and *C*). A549 cells stably expressing PME-1 targeting shRNA (shPME-1) and shPME-1-resistant FLAG-PME-1 (A549 FLAG-PME-1) were generated to confirm the inhibitory effect of SB218078 on PME-1/PP2Ac PPI by immunoprecipitation. A549 FLAG-PME-1 cells were treated with SB218078 and the FLAG M2 beads were utilized to immunoprecipitate FLAG-PME-1. SB218078 treatment decreased PP2Ac that was associated with FLAG-PME-1 (Fig. 2, *D* and *E*). SB218078 did not affect the luminescent intensity of *in vitro* NanoBiT assay for PME-1/PP2Ac PPI and LSmBiT (Figs. 2*F* and S2*E*), and the protein expression of PP2Ac and PME-1 (Fig. 2, *G*–*I*). These data suggest that SB218078 dissociates PME-1 from PP2Ac by altering intracellular signaling.

Although the primary target of SB218078 is CHK1 (IC_50_ 15 nM), it also weakly inhibits CDK1 activity (IC_50_ 250 nM). To confirm the involvement of CHK1, A549 PME-1/PP2Ac cells were treated with a CDK1 inhibitor alsterpaullone (26), a CHK1-specific inhibitor rabusertib (27), and a CHK1/CHK2 inhibitor prexasertib (28) (Fig. 2, *J*–*L*). Rabusertib and Prexasertib, but not alsterpaullone, suppressed the PME-1/PP2Ac association, supporting the idea that CHK1 plays a crucial role in the PME-1 and PP2Ac association.

### CHK1 phosphorylates PP2Ac and promotes PME-1/PP2Ac association

Since PP2Ac is the substrate of PME-1, the methylation status of PP2Ac may affect PME-1/PP2Ac association. The C-terminus leucine residues of type 2A protein phosphatases (PP2Ac, PP4c, and PP6c) undergo methylation by LCMT1 (29). Although there was a possibility that the anti-demethylated (deM) PP2Ac antibody may cross-react with PP4c and PP6c due to the similarity in the C-terminal sequences, the specificity of the antibody was confirmed (Fig. S3*A*). SB218078 treatment did not affect the PP2Ac methylation levels (Fig. S3, *B* and *C*), suggesting that SB218078-induced dissociation of PME-1 from PP2Ac was not due to the changes in PP2Ac methylation levels. Therefore, we speculated that PP2Ac and/or PME-1 phosphorylation was involved in the CHK1-induced PME-1/PP2Ac association.

**Figure 3:**
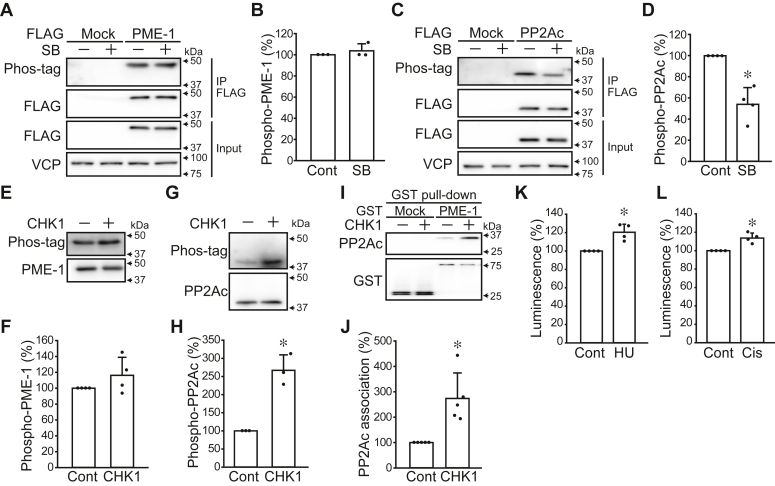
**CHK1 phosphorylates PP2Ac and promotes PME-1/PP2Ac association.***A*–*D*, A549 FLAG-PME-1 cells (*A* and *B*) and A549 FLAG-PP2Ac cells (*C* and *D*) were treated with SB218078 (1 μM) for 24 h and immunoprecipitation was performed using FLAG-M2 beads. Phos-tag biotin-HRP was used to analyze phosphorylation levels of PP2Ac and PME-1. Representative images (*A* and *C*) and quantitative data (*B* and *D*) are shown. *E*–*H*, *in vitro* kinase assay. Recombinant PME-1 and PP2Ac were treated with active CHK1. Phosphorylation levels of PME-1 (*E* and *F*) and PP2Ac (*G* and *H*) were analyzed by Phos-tag biotin-HRP. *I* and *J*, GST pull-down assay. Recombinant PP2Ac was treated with or without CHK1 and incubated with recombinant GST and GST-PME-1 on beads. Representative images (*I*) and quantitative data (*J*) are shown. *K* and *L*, A549 PME-1/PP2Ac cells were treated with hydroxyurea (HU, 2 mM) (*K*) and cisplatin (2 μM) (*L*) for 24 h and analyzed the luminescent intensity of PME-1/PP2Ac PPI. ∗*p* < 0.05. Data points are independent biological replicates.

A549 FLAG-PME-1 cells and A549 cells stably expressing FLAG-PP2Ac (A549 FLAG-PP2Ac) were treated with SB218078, and the phosphorylation levels of immunoprecipitated FLAG-PME-1 and FLAG-PP2Ac were analyzed using Phos-tag biotin-HRP. While SB218078 did not affect the phosphorylation level of PME-1 (Fig. 3, *A* and *B*), it decreased the PP2Ac phosphorylation level (Fig. 3, *C* and *D*). *In vitro* kinase assay revealed that CHK1 directly phosphorylates PP2Ac but did not affect the phosphorylation level of PME-1 (Figs. 3, *E*–*H* and S3*D*). GST-pulldown assay using GST-PME-1 and His-PP2Ac showed that CHK1-induced phosphorylation of PP2Ac promotes the association with PME-1 (Figs. 3, *I* and *J* and S3*E*). Treatment A549 PME-1/PP2Ac cells with CHK1 activating reagents, hydroxyurea and cisplatin, promotes the association of PME-1 with PP2Ac (Fig. 3, *K*–*L*). These data indicate that CHK1 phosphorylates PP2Ac to enhance PME-1 association.

### CHK1 phosphorylates PP2Ac at multiple sites including Thr219

**Figure 4:**
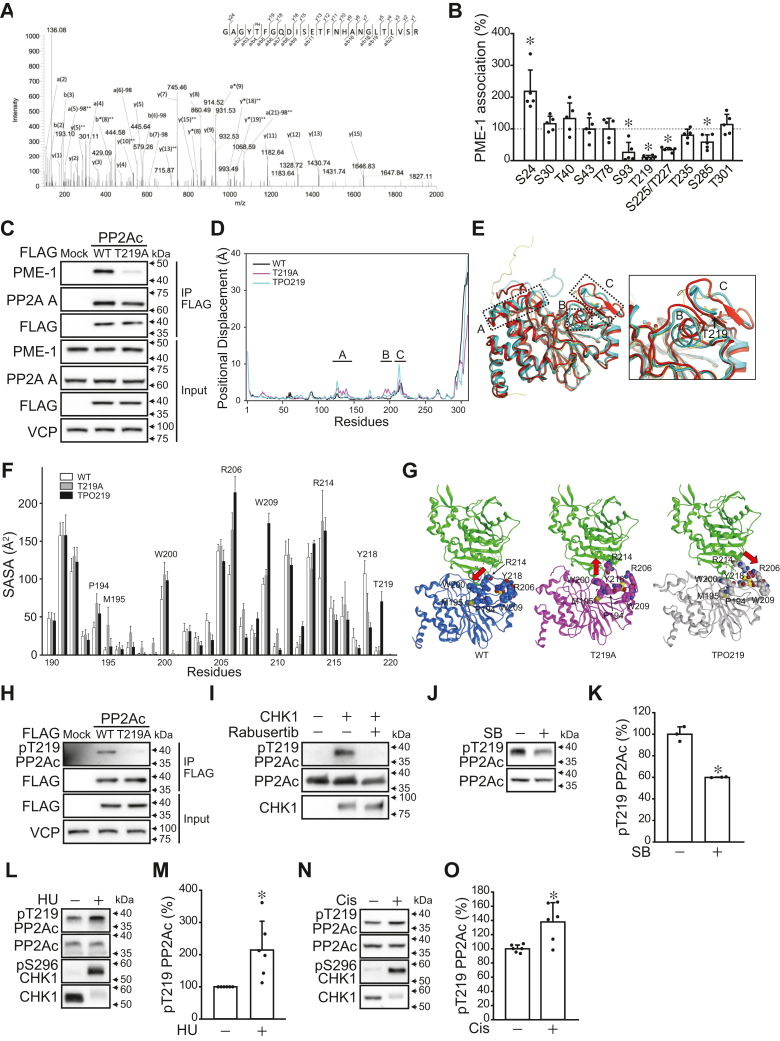
**CHK1 phosphorylates PP2Ac at multiple sites, including Thr219.***A*, phospho-mass spectrometry was performed to identify the phosphorylation sites of PP2Ac by CHK1. The representative LC/MS/MS spectrum shows phosphorylation at Thr219 of PP2Ac. *B* and *C*, 293T cells were transiently expressed FLAG-tagged PP2Ac mutants, and the association with PME-1 was analyzed using immunoprecipitation. Quantitative data (*B*) and representative images for PP2Ac T219A mutant (*C*) are shown. Representative images of other mutants can be found in Fig. S4. *D*–*G*, molecular dynamics simulation was carried out for PP2Ac wild-type (WT), T219A mutant, and Thr219 phosphorylated-type (TPO219). *D*, positional displacement for PP2Ac WT (*black*), T219A (*magenta*), and TPO219 (*cyan*). Three characteristic peaks (regions *A*–*C*) were indicated. *E*, structural comparison between PP2Ac WT and T219A. The initial structure for MD simulation, 1-μsec structure of WT and T219A are shown in *yellow*, *cyan*, and *red*, respectively. The *dotted boxes* indicate the regions (*A*–*C*) of the three characteristic peaks. The two Mn^2+^ are shown by ball representation. The inset is an enlarged view of the regions (*B* and *C*). The amino acid residues at 219 are indicated by *stick representation*. *F*, solvent accessible surface area (SASA) of regions (*B* and *C*). The averaged SASA per residue for the last 200 ns MD trajectories of the PP2Ac WT (*white*), T219A (*gray*), and TPO219 (*black*) is shown. *G*, the modeled complex of the simulation structure of the PP2Ac binding to PME-1. The 1-μsec MD simulation structures of PP2Ac WT (*blue*), T219A mutant (*magenta*), and TPO219 (*grey*) are superimposed on the X-ray structure of the PP2Ac-PME-1 complex (PDB ID: 3C5W). Eight residues (P194, M195, W200, R206, W209, R214, Y218, and T219) are shown in space-fill representation. *Red arrows* indicate the orientation of R219. *H*, FLAG-tagged PP2Ac WT and T219A mutant were immunoprecipitated from 293T cells and the specificity of the anti-phospho-Thr219 PP2Ac antibody was analyzed by immunoblotting. *I*, *In vitro* kinase assay was performed to phosphorylate recombinant PP2Ac by CHK1 with or without CHK1 inhibitor rabusertib (LY2603618, 9 μM). Anti-phospho-Thr219 PP2Ac antibody was used for immunoblotting. *J*–*O*, A549 cells were treated with SB218078 (5 μM, 8 h) (*J* and *K*), hydroxyurea (HU, 2 mM, 24 h) (*L* and *M*), and cisplatin (2 μM, 24 h) (*N* and *O*). Immunoblotting was performed to analyze the Thr219 phosphorylation levels of PP2Ac. Representative images (*J*, *L*, and *N*) and quantitative data (*K*, *M*, and *O*) are shown. ∗*p* < 0.05. Data points are independent biological replicates.

Phospho-mass spectrometry following *in vitro* kinase assay was performed to identify the CHK1-induced phosphorylation sites on PP2Ac. Phospho-peptides were not detected for CHK1-untreated recombinant PP2Ac. On the other hand, 12 phospho-sites (Ser24, Ser30, Thr40, Ser43, Thr78, Ser93, Thr219, Ser225, Thr227, Thr235, Ser285, and Thr301) were detected for CHK1-treated recombinant PP2Ac (Fig. 4*A* and Supplemental File 2). The FLAG-tagged alanine mutants of these sites were generated, and immunoprecipitation was performed to analyze the association with endogenous PME-1. Several mutants (S93A, T219A, S225/T227A, S285A) showed decreased PME-1 association (Figs. 4, *B* and *C* and S4*A*). S93A and T235A affected the association with PP2AA subunit, suggesting a potential influence on the overall structure of PP2Ac (Fig. S4, *A* and *B*). Notably, the PP2Ac T219A mutation resulted in an almost complete loss of PME-1 binding without affecting the binding to the A subunit and protein stability (Figs. 4, *B* and *C* and S4, *B*–*D*).

Thr219 of PP2Ac is located at the base of the loop structure that binds to PME-1 (Fig. S5*A*). Since it is not in direct contact with PME-1, the impact of the PP2Ac T219A mutation and phosphorylated Thr219 on the PP2Ac structure was analyzed by molecular dynamics simulation. The initial structure of PP2Ac was built as described in Experimental procedures (Fig. S5*B*). Root mean square deviation (RMSD) and positional displacement of the MD trajectories for the PP2Ac WT, T219A mutant, and phosphorylated-type (TPO219) were analyzed at the backbone level. The RMSD analysis revealed that the C- and N-terminal regions of these three PP2Ac were significantly altered from the initial structure because these regions do not have secondary structures. On the other hand, the rest part of PP2Ac (residue 10–290) remained stable (Fig. S5*C*). Positional displacement was analyzed by superimposing the MD trajectories on the initial structure (residues 10–290) and calculating the average displacement of the Cα atoms of each residue at each MD trajectory (last 200 ns) from the initial structure. There are three characteristic peaks (regions A, B, and C) in the residues 10 to 290 (Figs. 4, *D* and *E* and S5*D*). In region A (around residues 125–140), the conformational change in the T219A mutant and TPO219 was caused by the interactions with the C-terminal tail and was unlikely to result from the mutation. In region B (around residues 193–198), the α-helix structure deviates from the initial structure in the T219A mutant, while such deviation was not observed in the WT and TPO219. Region C (around residues 206–217) is a flexible region containing a loop structure in addition to the β-sheet structure. Hence, all three structures showed large deviations in this region. More precisely, the structural change in the T219A mutant and TPO219 is greater than in the WT, presumably due to the introduction of the T219A mutation and phosphorylated Thr219 in this region. Regions B and C are in close proximity and form a dynamic interaction network. Therefore, the conformational change in the α-helix in region B, which appeared only in the T219A mutant, is presumably due to the mutation of Thr219.

Therefore, we analyzed solvent-accessible surface area (SASA) values of regions B and C (Fig. 4*F*). Six residues (P194, M195, W200, R206, R214, and Y218) were identified with differences more than 30 Å^2^ between WT and T219A. In five of the six residues, except for Y218 (next to the mutant residue), the SASA values of the T219A mutant are greater than those of the WT. Additionally, five residues (R206, W209, R214, Y218, and phosphorylated T219) were identified with differences of more than 30 Å^2^ between WT and TPO219. In four of these five residues, except for Y218, the SASA values of the phosphorylated type are greater than those of the WT. These findings indicate that the directionality of side chains also altered along with the conformational changes in the backbone.

The conformations of the key six or five residues were analyzed by superimposing the 1-μsec MD simulation structures on the X-ray structure of the PP2Ac-PME-1 complex (PDB ID: 3C5W (20)) (Fig. 4*G*). The comparison of the WT and T219A mutant indicates that the side chains of the six residues are oriented differently. Among them, the structural change in R214 is substantial, and R214 in the T219A mutant is in conformational collision with PME-1, which would affect binding to PME-1. The comparison of the WT and TPO219 also showed that the side chains of five residues have different conformations. In the TPO219, R214 formed salt bridges with the phosphorylated Thr219, which was not observed in the WT and T219A mutant (Fig. S5*E*). The formation of the salt bridge stabilizes the conformation of R214. Consequently, the surrounding structure of R214 does not interfere with the binding to PME-1 and may enhance its binding affinity. These results suggest that modification of Thr219 residue may play a pivotal role in the association with PME-1.

To confirm that CHK1 phosphorylates PP2Ac at Thr219, an anti-phospho-Thr219 PP2Ac antibody was generated. Dot blot analysis showed that this antibody recognized phospho-Thr219 peptide, but not non-phospho-Thr219 peptide, which was blocked by the addition of phospho-Thr219 peptide during the antibody reaction (Fig. S6*A*). Moreover, FLAG-PP2Ac WT, but not T219A, immunoprecipitated from 293T cells was recognized by this antibody, confirming the specificity of the antibody (Fig. 4*H*). This antibody recognized recombinant PP2Ac treated with CHK1, which was blocked by the addition of a CHK1 inhibitor (Fig. 4*I*). Treatment A549 cells with SB218078 decreased the Thr219 phosphorylation levels of PP2Ac, while the treatment with hydroxyurea and cisplatin, which activates CHK1, increased the Thr219 phosphorylation levels (Fig. 4, *J*–*O*). SB218078 decreased the PP2Ac Thr219 phosphorylation levels in PC9 cells, but not in MIA PaCa-2 and HCT116 cells (Fig. S6, *B* and *C*). Moreover, hydroxyurea increased Thr219 phosphorylation levels of PP2Ac in PC9, MIA PaCa-2, and HCT116 (Fig. S6, *D* and *E*). These data indicate that CHK1 phosphorylates PP2Ac at Thr219 in cells.

### PP2A/PME-1 axis regulates CHK1 activity

**Figure 5:**
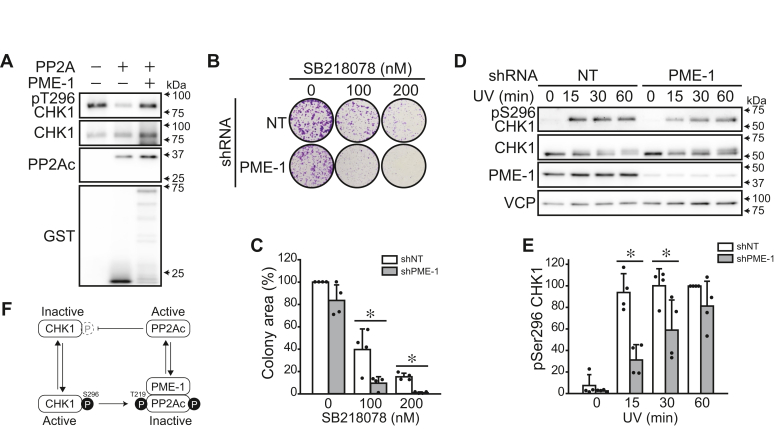
**PP2A/PME-1 axis regulates CHK1 activity.***A*, *in vitro* phosphatase assay was performed to analyze CHK1 dephosphorylation by PP2Ac. GST-PME-1 S156A mutant which lacks methyl-esterase activity was added to inhibit PP2Ac. *B* and *C*, a colony formation assay was performed for A549 cells stably expressing non-target shRNA (shNT) and PME-1-targeting shRNA (shPME-1). Cells were treated with the indicated dose of SB218078. *D* and *E*, A549 cells stably expressing shNT and shPME-1 were exposed to UV radiation. Immunoblotting was performed to analyze CHK1 phosphorylation levels. Representative images (*D*) and quantitative data (*E*) are shown. *F*, model of CHK1/PME-1/PP2Ac tripartite relationship. ∗*p* < 0.05. Data points are independent biological replicates.

Our data indicates that CHK1 phosphorylates PP2Ac to associate with PME-1, even without stimuli that would promote CHK1 activity. Finally, we explored the physiological significance of this regulatory mechanism. Since PP2A dephosphorylates CHK1 (24, 25), we hypothesized that CHK1 maintains its basal activity by suppressing PP2Ac through PME-1 association. CHK1 is activated by the phosphorylation of Ser317 and Ser345 followed by the auto-phosphorylation of Ser296 (30). *In vitro* phosphatase assay using recombinant CHK1 and PP2Ac revealed that PP2Ac dephosphorylated CHK1 at Ser296 (Fig. 5*A*). This dephosphorylation was blocked by the addition of recombinant PME-1 S156A mutant (lacks methylesterase activity). A549 cells stably expressing non-target shRNA (shNT) and shPME-1 were generated to test the combination effects of CHK1 inhibitor and PME-1 KD. SB218078 showed dose-dependent inhibition in the colony-formation assay; PME-1 KD did not affect the colony formation under untreated conditions but increased sensitivity to SB218078 (Fig. 5, *B* and *C*). In A549 cells expressing shNT, Ser296 phosphorylation of CHK1 was rapidly induced after UV irradiation (Fig. 5, *D* and *E*). This increase in phosphorylation was delayed when PME-1 was knocked down but reached the same level at 60 min after stimulation. A similar delay in the UV-induced CHK1 Ser296 phosphorylation was observed when PP2Ac was overexpressed (Fig. S7, *A* and *B*). These data suggest that the phosphorylation of PP2Ac by CHK1 contributes to the maintenance of basal activity of CHK1 and its rapid activation upon DNA injury by promoting the binding of PP2Ac to PME-1.

## Discussion

Kinase inhibitors are widely used in clinical oncology. However, challenges such as drug resistance and the presence of non-responsive cancers have arisen. The activation of phosphatases represents another approach to inhibiting excessive phosphorylation. In particular, the reactivation of PP2A, a critical cancer suppressor, is gaining attention as a novel anticancer strategy (4, 5). PP2A is a holoenzyme, and activation of a subset of the PP2A trimer provides anticancer effects. To achieve this, approaches include directly promoting the assembly of specific PP2A trimers or inhibiting the binding between PP2A holoenzyme and the PP2A inhibitory proteins such as SE translocation (SET) (31, 32, 33). PME-1 is also a potential drug target since it contributes to the malignant transformation of cancer, but a lack of knowledge about the complicated functions of PME-1 has hindered this. Because there are cases where the results of PME-1 knockdown are not reproduced by the PME-1 methylesterase inhibitor ABL127 (18), the function as a PP2A inhibitor is thought to contribute to cancer promotion. However, previous *in vitro* experiments showed that ABL127 inhibits PME-1/PP2Ac association (7, 22). The results of the present study strengthen these findings by showing that ABL127 inhibits PME-1/PP2Ac PPI even in living cells. Insights from recent structural analyses may serve as the key to resolving this contradiction: PME-1 not only binds to the active site of PP2Ac but also directly interacts with regulatory subunits such as B56γ, inhibiting the activity of PP2A trimer (21). Since PME-1 binds to B56γ *via* short linear motifs (SLiMs), ABL127, which binds to the enzyme active site, would not inhibit the binding of PME-1 to B56γ. Although further studies are needed, compounds that inhibit the SLiMs-mediated association of PME-1 with the B subunits may be promising for anticancer effects.

The regulation of PME-1 as a PP2A inhibitory protein had remained unclear. High-throughput screening assays utilizing the NanoBiT system prove effective in investigating the molecular mechanisms of PPI. Of the 399 compounds tested, this study focused on a CHK1 inhibitor SB218078, but it is worthwhile to analyze the signal pathways regulated by other compounds. Although SB218078 has weak inhibitory effects on CDK1, our data supports the idea that CHK1 directly governs PME-1/PP2Ac PPI. Cell-based assay and *in vitro* kinase assay showed that PP2Ac, but not PME-1, is a substrate of CHK1. In contradiction to our results, a phospho-mass spectrometry analysis has previously shown that CHK1 phosphorylates PME-1 at Ser15 (34). Phos-tag detects all phosphorylation on PME-1, potentially obscuring changes in Ser15 phosphorylation levels. The lack of commercially accessible antibodies targeting phospho-Ser15 PME-1 has impeded the validation of this aspect. However, since phospho-Ser15 of PME-1 is a substrate of PP2A (35), it is unlikely that phosphorylation of this site promotes the function of PME-1 as PP2A inhibitory protein.

The phospho-proteomic analysis revealed that CHK1 phosphorylates PP2Ac at multiple sites, and mutation analysis showed that several of them are potentially involved in PME-1/PP2Ac association. The PP2Ac T219A mutation resulted in an almost complete loss of PME-1 association, and the phosphorylation levels of Thr219 in cells were decreased and increased by CHK1 inhibitors and activators, respectively. Similar results were observed in multiple cell lines. SB218078 did not suppress Thr219 phosphorylation levels of PP2Ac in MIA PaCa-2 and HCT116 cells, probably due to the low activity of the phosphatase that dephosphorylates this site or the involvement of other kinases. The molecular dynamics simulation suggested that the modification of the Thr219 residue of PP2Ac may change the structure of the contacting surface with PME-1. The substitution of alanine at multiple sites individually reduced binding, suggesting that the CHK1-induced association of PME-1/PP2Ac may involve multiple phosphorylation events in a complex manner. Therefore, comprehending the structural overview of how CHK1 facilitates the binding between PME-1 and PP2Ac is not straightforward.

Several studies have demonstrated that PP2A dephosphorylates CHK1 (24, 25), a finding corroborated by *in vitro* phosphatase assay conducted in this study. Therefore, CHK1 and PP2Ac are in an enzyme-substrate relationship with each other (Fig. 5*F*). Although the present study does not fully unravel the significance of the interplay between CHK1 and PP2Ac, our results suggest one potential aspect. Recombinant PME-1 hindered CHK1 dephosphorylation by PP2Ac, suggesting that CHK1 promotes PME-1/PP2Ac association to escape from the PP2A-mediated inactivation and maintain its basal activity. CHK1 plays a pivotal role in DNA damage repair from exogenous stimulation such as UV, but it is also involved in the normal cell cycle progression (36). Phosphorylation of CHK1 was barely detectable in unstimulated cells. However, the reduction in PP2Ac Thr219 phosphorylation levels and PME-1/PP2Ac PPI following CHK1 inhibition provide evidence that CHK1 activity exists to a certain extent. In the present study, suppression of PME-1 expression sensitizes cells to SB218078. These data are supported by the drug sensitivity screening showing the combination effects of PME-1 KD and CHK1 inhibitor AZD7762 in A549 and HeLa cells (37, 38). These observations suggest the potential for a combined effect with CHK1 inhibitor and the inhibition of CHK1 activity by PP2Ac released from PME-1. The positive correlation between the CHK1 and PP2Ac/PME-1 protein levels may reflect the significance of the tripartite relationship in resting cells. PME-1 KD delayed the CHK1 phosphorylation immediately after UV irradiation, also suggesting that PP2Ac suppresses CHK1 in the quiescent cells. On the other hand, as time passed after UV irradiation, the difference in CHK1 phosphorylation levels between shNT and shPME-1 diminished. Although the stimuli that activate CHK1 increased the PP2Ac Thr219 phosphorylation levels, the CHK1/PME-1/PP2Ac axis may not play a pivotal role in DNA damage response caused by exogenous stimuli.

Dysregulation of PP2Ac methylation has been implicated in neurodegenerative diseases such as Alzheimer's disease (AD). Aging causes aberrations in the PP2Ac methylation regulatory mechanism in the brains of cynomolgus monkeys (39). The expression of PME-1 is increased in patients with AD, and the PME-1 overexpression in the brains of AD model mice worsens symptoms (40, 41). However, it cannot be ruled out that the function of PME-1 as a PP2A inhibitory protein is also involved in this. Hu *et al.* reported that CHK1 inhibitors restored PP2A activity and ameliorated AD pathogenesis and cognitive dysfunction in the mouse model (42). In their model, CHK1 inhibitors activate PP2A by suppressing the PP2A inhibitory protein CIP2A expression. There is also a possibility that the dissociation of PME-1 from PP2Ac is involved in this process.

## Experimental procedures

### Cell cultures

A549, 293T, PC9, MIA PaCa-2, and HCT116 cells were obtained from RIKEN BRC and JCRB Cell Bank. A549, 293T, MIA PaCa-2, and HCT116 cells were grown in DMEM containing 10% fetal bovine serum (FBS) and 1× anti-biotic/antimycotic (Life Technologies). PC9 was grown in RPMI containing 10% FBS and 1× anti-biotic/antimycotic. Contamination of *mycoplasma* was tested using MycoAlert (Lonza) and cells were occasionally treated with plasmocin (Nacalai tesque) to prevent *mycoplasma* contamination.

### Plasmids, transfection, and virus production

pLVSIN nFLAG3 human PP2Ac, PME-1, and PP6c were previously described (7, 43). pLVSIN nFLAG3 human PP2Ac S24A, S30A, T40A, S43A, T78A, S93A, T219A, S225A/T227A, T235A, S285A, and T301A, pcDNA3 nFLAG2 PP2Ac and PP4c, pLVmCherry for non-target shRNA (shNT) and PME-1 targeting shRNA (shPME-1) were generated using InFusion HD Cloning Kit (Takara Bio). The sequences of shRNA are as follows: shNT, 5′-CAACAAGATGAGAGCACCA-3′, shPME-1#1, 5′-GGGCGATACATCTGAGTTCA-3′.

To produce lentiviruses, pLVSIN, a packaging plasmid (psPAX2), and a coat protein plasmid expressing vesicular stomatitis virus G protein (pMD2.G) were diluted in Opti-MEM containing Polyethylenimine MAX (PEI, Polysciences). The mixture was added to Lenti-X 293T cells (Takara Bio) Medium was replaced with fresh medium after 8 h, and cells were cultured for 48 h. The viral supernatants were filtered using a 2.2 μm filter (Millipore) and added to cells.

### Protein-protein interaction assay using NanoBiT system

pBiT plasmids (Promega: pBiT1.1C, pBiT1.1N, pBiT2.1C, and pBiT2.1N) for PP2A and PME-1 were generated using InFusion HD Cloning Kit. Plasmids were transfected into 293T cells, and luminescent intensity was analyzed using Nano-Glo Live Cell Assay System (Promega) and GloMax Explorer System (Promega) set at 37 °C.

The sequences for N-terminal SmBiT-tagged PP2Ac and C-terminal LgBiT-tagged PME-1 were PCR amplified and inserted into pLVnB plasmid using InFusion HD Cloning Kit. LSmBiT was designed as a protein consisting of LgBiT and SmBiT connected by a linker sequence (GSSGGGGSGGGGSSGGAQGNSV). A549 cells were infected with lentivirus vectors to express these proteins stably.

For the screening with *in vivo* NanoBiT assay, 2 × 10^4^ cells of A549 stably expressing LgBiT-PME-1 and SmBiT-PP2Ac (A549 PME-1/PP2Ac) were seeded on 96 well plates. After 24 h, compounds (Supplemental File 1) were applied to the well (5 μM) and incubated for 24 h. The luminescence was analyzed using the Nano-Glo Live Cell Assay System and normalized with the cell viability analyzed using CellTox Green (Promega). The luminescent intensity was expressed as a percentage with the DMSO-treated cells at 100%.

For the *in vitro* NanoBiT assay, 1 × 10^6^ cells of A549 PME-1/PP2Ac were lysed in 1 ml of CHAPS lysis buffer (40 mM HEPES, 150 mM NaCl, 2 mM EDTA, 0.3% CHAPS, 10 mM disodium glycerophosphate, 10 mM sodium pyrophosphate, one tablet/50 ml Roche Complete, pH 7.4). After centrifugation at 15,000 rpm for 15 min, supernatants were collected and used for the following experiments. Compounds were added and incubated at 4 °C for 1 h. The luminescence was analyzed using the Nano-Glo Live Cell Assay System. The luminescent intensity was expressed as a percentage with the DMSO-treated wells as 100%.

### Immunoblotting

Immunoblotting was performed as described previously (39). Briefly, cells were lysed in CHAPS lysis buffer containing Roche Complete protease inhibitor mixture and PhosSTOP or a buffer containing 50 mM Tris-HCl (pH 8.0), 5 mM EDTA, 5 mM EGTA, 1% Triton X100, 1 mM Na_3_VO4_4_ 20 mM sodium pyrophosphate, and Roche Complete protease inhibitor mixture. The protein concentration of cell lysates was analyzed using a DC protein assay kit (Bio-Rad). The equal amounts of proteins were applied to SDS-PAGE and transferred onto a nitrocellulose membrane (Wako). The membranes were treated with 0.5% skim milk or EzBlock Chemi (ATTO) for 1 h and primary antibodies (0.5–1 μg/ml) in a Tris-buffered saline with 0.05% Tween 20 overnight. After the treatment with biotin-labeled secondary antibody for 1 h, immunoreactive bands were visualized using AMERSHAM ImageQuant 800 (Cytiva) and LuminoGraph II EM (ATTO), and band densities were quantified using ImageJ (National Institutes of Health). Valosin-containing protein (p97/VCP) and actin were used as the loading control. The PP2A methylation levels were analyzed using a base-treatment method as previously described (39). The methylation levels were determined by calculating the ratio of band densities of anti-demethylated PP2A antibodies between the NaOH-treated and untreated samples.

Antibodies were obtained from the following supplier: anti-p97/VCP (Gene Tex, GTX113030), anti-FLAG (F7425, SIGMA), anti-PME-1 (SC-25278, Santa Cruz,), anti-PP2Ac (07–324, Millipore,), anti-demethylated PP2Ac (SC-13601, Santa Cruz), anti-CHK1 (2360, Cell Signaling), anti-LgBiT (N7100, Promega), anti-pSer296 CHK1 (2349, Cell Signaling), and anti-GST (2624, Cell Signaling). Anti-SmBiT antibody was provided by Promega. Anti-phospho-Thr219 PP2Ac antibody was generated by SCRUM Inc. The phosphorylated peptide Cys+PRGAGY(pT)FGQDIS was used to immunize rabbits. The antibody was purified from the antiserum by using phosphorylated peptide-conjugated resin, and further purified by passing it through non-phosphorylated peptide-conjugated resin.

### Immunoprecipitation

Cells were lysed in a CHAPS lysate buffer, and the protein concentration was analyzed using a DC protein assay kit. FLAG M2 beads (SIGMA) were added to the samples and incubated at 4 °C for 30 min. After washing the beads with CHAPS Wash Buffer (CHAPS lysate buffer without Roche Complete), proteins were eluted using 3× FLAG peptide (SIGMA). The association of proteins was analyzed by immunoblotting.

### *In vitro* kinase assay

Recombinant His-PP2Ac was generated as previously described (7). GST-CHK1 was purchased from SignalChem. His-PME1 was expressed in BL21(DE3) *E. coli* cells. The cells were sonicated, and after centrifugation, the clarified supernatant was applied onto a HisTrap column (Cytiva) preequilibrated with a buffer containing 20 mM imidazole, 20 mM Tris-HCl (pH 8.0), and 500 mM NaCl. The resin was washed with 10 column volumes by the same buffer. The proteins were eluted with a buffer containing 500 mM imidazole, 20 mM Tris-HCl (pH 8.0), and 500 mM NaCl. The proteins were concentrated and further purified by the size exclusion chromatography using a Superdex 200 column (Cytiva) preequilibrated with a buffer containing 25 mM HEPES (pH 7.5), and 150 mM NaCl.

1 μg of His-PP2Ac and His-PME-1 were added to the kinase assay buffer (50 mM Tris-HCl, 10 mM MgCl_2_, 0.1 mM EDTA, 0.005% Tween 20, 2 mM DTT, 0.4 mM ATP, pH 7.5) and incubated with or without GST-CHK1 (0.15 μg) at 30 °C, 200 rpm for 1 h. SDS sample buffer (50 mM Tris-HCl, 0.4% SDS, 6% β-mercaptoethanol, 2% glycerol, pH 6.8) was added to terminate the reaction. The phosphorylated PP2Ac and PME-1 were detected using Phos-tag biotin BTL-104 (Wako).

### GST pull-down assay

GST-PME-1 and GST were generated as previously described (7). GST-PME-1 and GST were incubated with Glutathione Sepharose 4B beads (Cytiva) in GST buffer (100 mM Tris-HCl pH 8.0, 200 mM NaCl) 1 h at room temperature. After washing the beads with GST wash buffer (GST buffer with 0.5% Triton X-100 and 1 mM EDTA), His-PP2Ac treated with or without CHK1 was added and incubated for 1 h at room temperature. After washing the beads, the associated proteins were eluted with SDS sample buffer and applied for immunoblotting.

### *In vitro* phosphatase assay

GST-PME-1 S156A, which lacks methyl-esterase activity, was generated as previously described (7). GST-CHK1 was treated with His-PP2Ac with GST or GST-PME-1 S156A in phosphatase assay buffer (50 mM Tris-HCl, 10 mM MgCl_2_, 0.02% Brij-35, 1 mg/ml BSA, pH 7.5) for at 30 °C, 200 rpm for 30 min. The reaction was terminated by adding an SDS sample buffer.

### Colony formation assay

1000 cells of A549 stably expressing shNT or shPME-1 were seeded on six well plates. The next day, compounds were added to the medium and cultured for another 9 days. Colonies were stained with Giemsa solution, and the colony area was analyzed using ImageJ.

### UV-irradiation

A549 cells were UV irradiated (10 mJ/cm^2^ of 254 nm UV-C) using UVP Crosslinker CL-3000 (UVP) and incubated for the indicated periods.

### Statistical analysis

The results are expressed as mean ± standard deviation. The Student’s *t* test and one-sample *t* test were used for comparisons between the two groups of parametric data and non-parametric data, respectively. For the comparison between three or more treatment groups to a single control group with a fixed value, analysis of variance (ANOVA) on the ranks test followed by the Dunnett test was performed. ANOVA test followed by the Tukey test was performed for comparisons between three or more groups with variance. All statistical analyses were performed using SigmaPlot (Systat Software), and statistical significance was set at *p* < 0.05.

## Data availability

All main text data are in this manuscript. Supplemental data are in the corresponding Supporting information document. Correspondence and requests for materials should be addressed to the corresponding author (t.ohama@yamaguchi-u.ac.jp).

## Supporting information

This article contains supporting information (44, 45, 46, 47, 48, 49, 50, 51, 52, 53, 54, 55, 56, 57).

## Conflict of interest

The authors declare that they have no known competing financial interests or personal relationships that could have appeared to influence the work reported in this paper.
