# Supplementary material for: The luciferase-based in vivo protein–protein interaction assay revealed that CHK1 promotes PP2A and PME-1 interaction
Source: J Biol Chem. 2024 Apr 6;300(5):107277. doi: 10.1016/j.jbc.2024.107277 (PMC11098961; doi:10.1016/j.jbc.2024.107277)
Supplement: Supporting information [file mmc3.pdf]

**Supporting Information**

## Methods

Reagents

Reagents were obtained from the following supplier: SB218078 (Cayman and Santa Cruz), LY2603618 (Cayman), hydroxyurea (Wako), cisplatin (Wako), okadaic acid (Cayman), ABL127 (SIGMA), cycloheximide (Wako), alsterpaullone (Adipogen), prexasertib (Cayman)

Phospho-mass spectrometry

CHK1-induced phosphorylation of recombinant PP2Ac was carried out, as mentioned in the in vitro kinase assay. PP2Ac was applied to SDS-PAGE, and each gel slice was extracted through in-gel digestion. The gel slices were diced into 1 mm pieces and subjected to a series of sequential steps: reduction with ten mM DTT at 56°C for one h, alkylation with 55 mM iodoacetamide at room temperature for 45 min in the dark, followed by digestion with trypsin (Thermo Fisher Scientific) at 37°C for 16 h. The resulting peptides were extracted using 1% trifluoroacetic acid and 50% acetonitrile solution. Phosphorylated peptides were enriched using the Titansphere Phos-TiO kit (GL science) and the High-Select™ Fe-NTA Phosphopeptide Enrichment Kit (Thermo Fisher Scientific) following the manufacturer's instructions. Initially, phosphopeptides were enriched using a TiO2-tipped column, and the flow-through fraction was further enriched for phosphopeptides with Fe-NTA. Both enriched fractions were combined and desalted using an in-house-made C18 stage-tip.

Mass spectra were acquired using an LTQ-Orbitrap Velos Pro (Thermo Fisher Scientific) coupled to a nanoflow UHPLC system (ADVANCE UHPLC; AMR Inc.) equipped with an Advanced Captive Spray SOURCE (AMR Inc.). Peptide mixtures were fractionated by C18 reverse-phase chromatography (3 μm, ID 0.075 mm × 150 mm, CERI). Elution occurred at a 300 nL/min flow rate with a linear gradient of 5–35% solvent B over 60 min. The composition of solution A and solution B was 2% acetonitrile, 0.1% formic acid, and 100% acetonitrile, respectively. The mass spectrometer was programmed for four scan events. The first event included a full MS scan from 350 to 1600 m/z using Orbitrap (resolution = 60,000), while the second to fourth events comprised data-dependent scans of the top three abundant ions obtained in the first scan using Orbitrap (resolution = 7,500). MS/MS spectra were automatically acquired from the highest peak in each scan, with the relative collision energy set to 35% HCD and an exclusion time of 90 seconds for molecules in the same m/z value range.

Raw files were subjected to a database search against the UniProt Human proteome database (downloaded on 2020.10.07) and the cRAP contaminant proteins dataset using the MASCOT program (version 2.6; Matrix Science) via Proteome Discoverer 2.4 (Thermo Fisher Scientific). The search criteria included carbamidomethylation of cysteine as a fixed modification, oxidation of methionine, acetylation of protein N-termini, and phosphorylation of serine, threonine, and tyrosine as variable

modifications. The number of allowed missed cleavage sites was set at 2.

Molecular Dynamics (MD) Simulations

Starting structures of wild-type, mutant-type (T219A), and phosphorylated-type PP2A catalytic subunits for molecular dynamics simulations were built based on the X-ray crystallographic structure (PDB ID: 2IE4 (44) Supplemental Fig. 4G). In the X-ray structure, the scaffolding subunit and okadaic acid were removed, and the missing N-terminal and C-terminal residues (residue 1-5, 294-309) of the PP2A catalytic subunit were added using the homology modeling technique with the Molecular Operating Environment (MOE) software (Chemical Computing Group Inc.). For the mutant and phosphorylated types, the threonine at residue 219 was replaced by alanine and phosphorylated threonine (TPO) using MOE software. Next, these wild-type and mutant structures were solvated in a rectangular box containing TIP3P water molecules (45). In addition, chlorine and sodium ions (0.15 M) were added to neutralize the system. Each system contained approximately 96,000 atoms.

The MD simulations were performed using the GROMACS version 5.0.6 software (46–50). The AMBER14SB force field (51) was used for proteins. The force field parameters for phosphorylated threonine (TPO) were those provided in the contributed force field parameters on the AMBER website (https://ambermd.org). The periodic boundary condition was applied to each system, and the temperature and pressure were kept constant using the V-rescale thermostat (52) and the Parrinello-Rahman barostat (53), respectively. The linear constraint solver (LINCS) algorithm (54) was applied to the covalent bonds, with an integration time step of 2.0 fs considered. The long-range Coulomb interactions were treated using the particle mesh Ewald method (55), and the direct space cutoff distance was set to 10.0 Å. The van der Waals interactions were calculated using a cutoff distance of 10.0 Å. Following the solved system energy minimization, the system was gradually heated to 298 K at 100 ps. Next, one μsec MD simulations were performed under an NPT ensemble (P = 1 bar and T = 298 K). The trajectories of each system were saved every 100 ps (10,000 conformations in each MD trajectory). For the analysis of the MD simulations, root mean square deviation (RMSD), positional displacement, and solvent accessible surface area (SASA) were computed. RMSD and positional displacement are performed by the CPPTRAJ module of AmberTools 20 (56). FreeSASA (57) was used to calculate SASA.

**Supplemental Figures**

Supplemental Figure 1

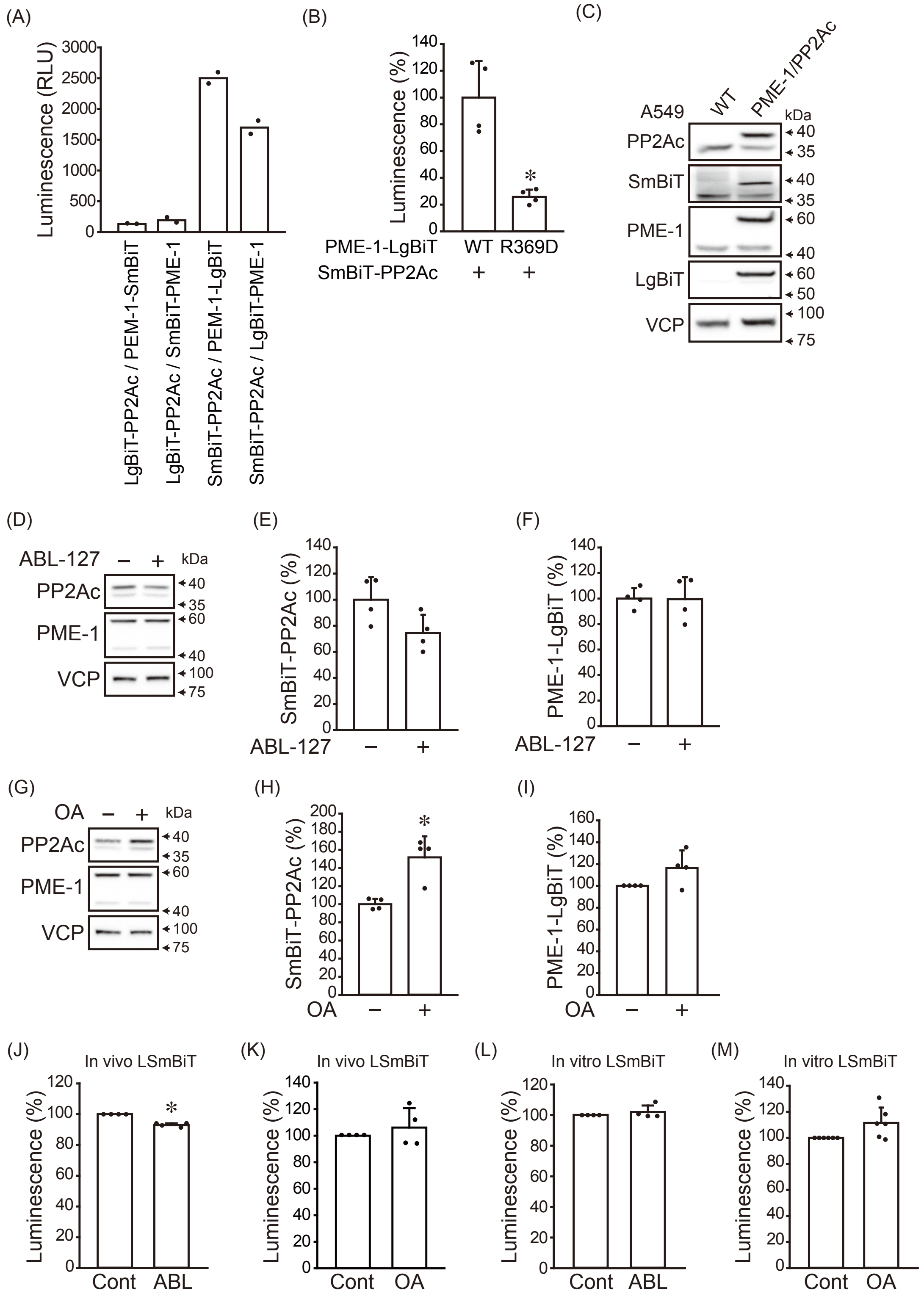

**Supplemental Figure 1**

(A–B) 293T cells were transiently expressed in the indicated combination of proteins, and the luminescence intensity was analyzed. (C) A549 cells were stably expressing LgBiT-PME-1 and SmBiT-PP2Ac (A549 PME-1/PP2Ac). Immunoblotting was performed to detect indicated proteins. The bands around 35 kDa recognized by the SmBiT antibody are nonspecific. (D–K) A549 cells stably expressing LSmBiT were treated with ten μM of ABL127 (D–F and J) and 100 nM of OA (G–I and K) for eight hours and analyzed immunoblotting (D–I) and luminescence intensity (J–K). (L–M) In vitro NanoBiT assay was performed to analyze the effect of 1 μM of ABL127 (L) and 100 nM of OA (M) on LSmBiT luminescence intensity. *: $P<0.05$. Data points are independent biological replicates.

Supplemental Figure 2

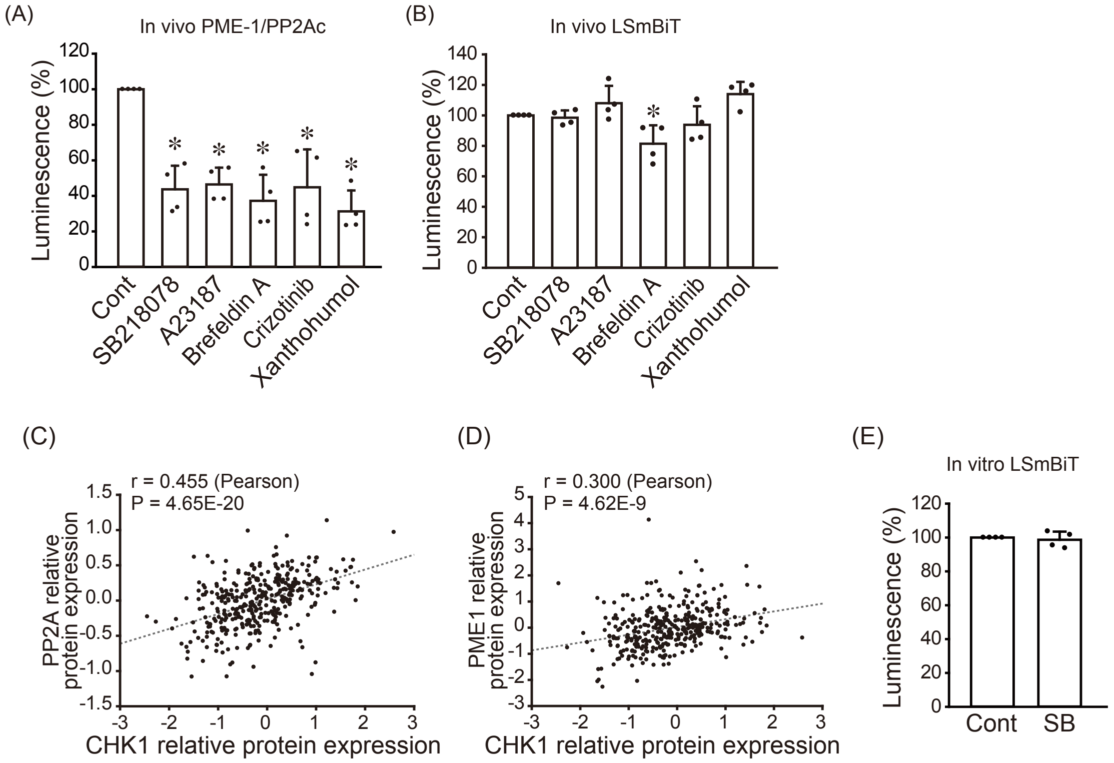


**Supplemental Figure 2**

(A–B) A549 PME-1/PP2Ac (A) and LSmBiT (B) cells were treated with 5 μM of indicated compounds, and luminescent intensity was analyzed. (C–D) Correlation analysis between CHK1 and PP2Ac (C) and CHK1 and PME-1 (D) in proteomics dataset using DepMap. (E) In vitro NanoBiT assay was performed to analyze the effect of 1 μM of SB218078 on LSmBiT luminescence intensity. *: $P<0.05$. Data points are independent biological replicates.

Supplemental Figure 3

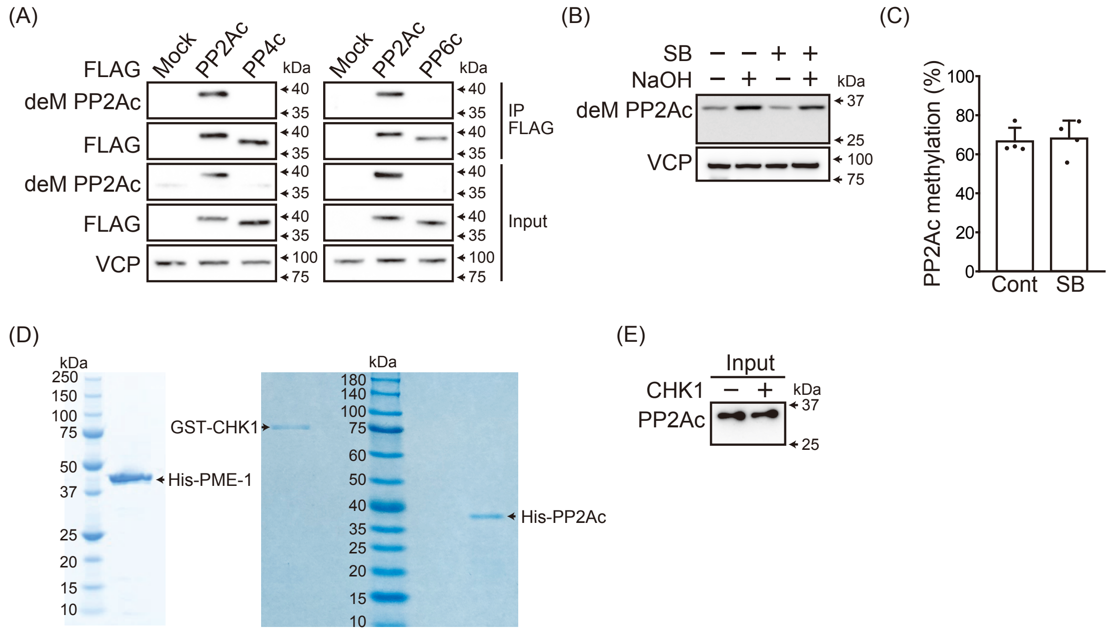


**Supplemental Figure 3**

(A) 293T cells were transiently expressed FLAG-tagged PP2Ac, PP4c, and PP6c. FLAL-tagged proteins were immunoprecipitated using FLAG-M2 beads, and immunoblotting was performed to confirm the specificity of the anti-demethylated (deM) PP2Ac antibody. (B–C) A549 cells were treated with SB218078 (0.5 μM) for 24 h. PP2Ac methylation levels were analyzed using a base-treatment method. Representative images (B) and quantitative data (C) are shown. (D) CBB staining of recombinant His-PME-1, GST-CHK1, and His-PP2Ac. (E) Recombinant PP2Ac was treated with or without active CHK1. Immunoblotting was performed to confirm that equal amounts of PP2Ac were applied to the GST-pulldown assay (Fig. 3I–J). Data points are independent biological replicates.

Supplemental Figure 4

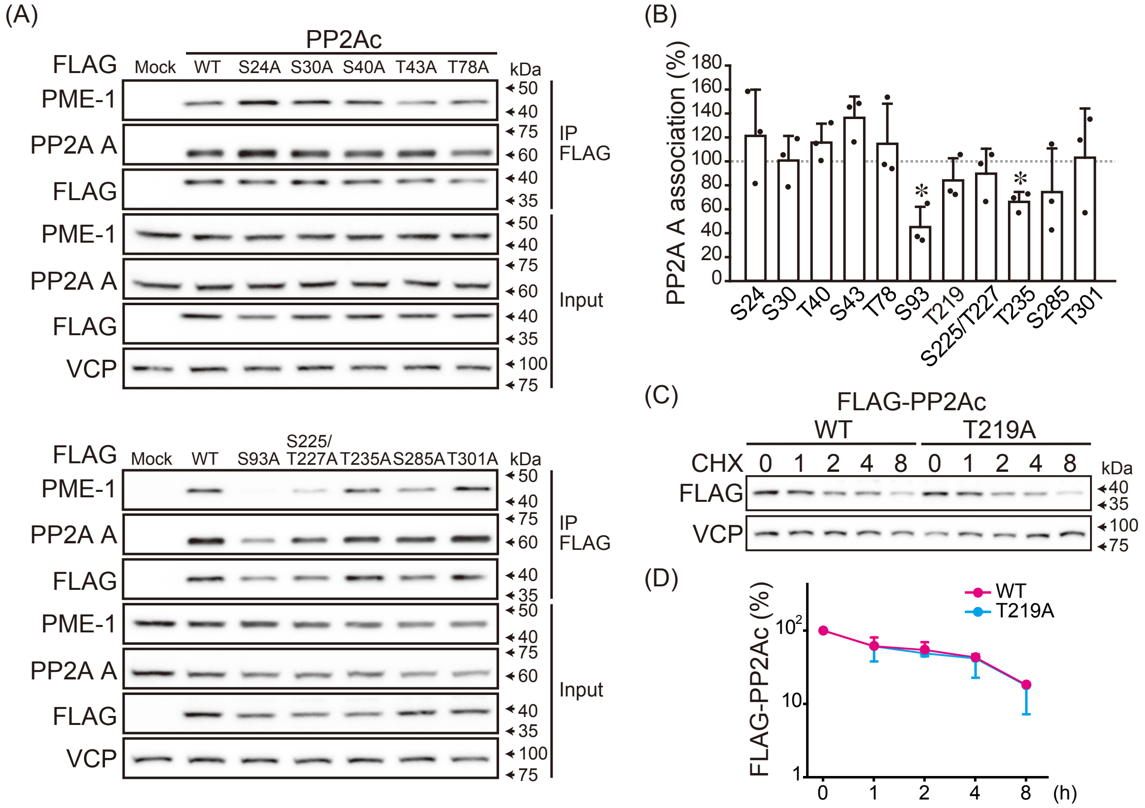


**Supplemental Figure 4**

(A–B) 293T cells were transiently expressed FLAG-tagged PP2Ac mutants, and the association with PME-1 and A subunit was analyzed using immunoprecipitation. Representative images (A) and quantitative data (B) are shown. (C–D) 293T cells were transiently expressed FLAG-tagged PP2Ac WT and T219A. Cycloheximide assay was performed to analyze the stability of proteins.

Supplemental Figure 5

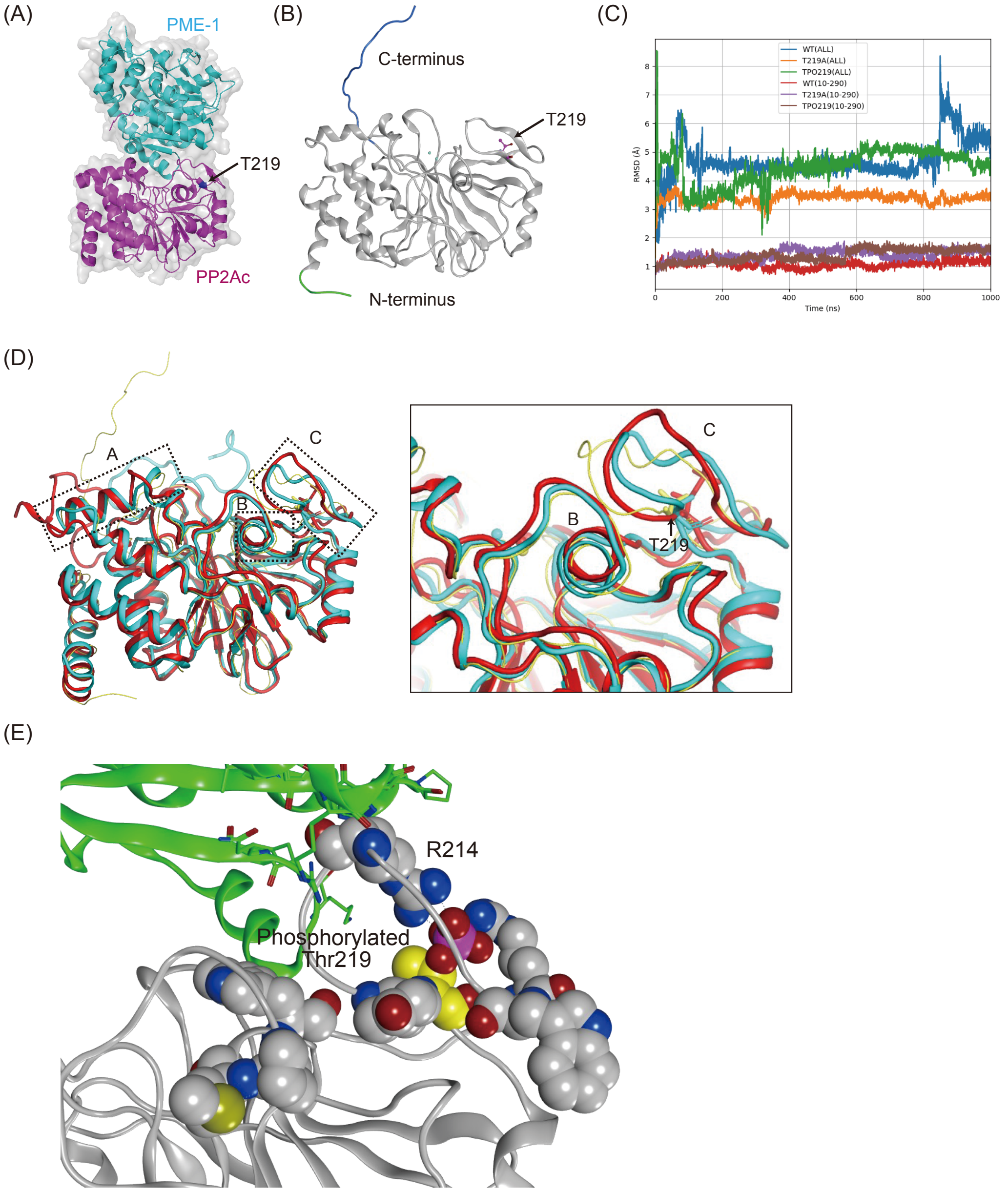

**Supplemental Figure 5**

(A) 3-D structure of PME-1 and PP2Ac complex (PDB: 3C5W). An arrow indicated the location of Thr219. (B) The initial structure of wild-type PP2A catalytic subunit. The green and blue strands indicate the modeled N-terminal and C-terminal regions. Neither region contains the secondary structures. For the PP2Ac T219A mutant, the threonine at residue 219 (magenta stick residue) was replaced by alanine. The cyan atoms are two $Mn^{2+}$ ions. (C) Time course of root mean square deviation (RMSD) for WT, T219A, and phosphorylated Thr219 (TPO219) PP2Ac. The RMSD values of the Cα atoms from the initial structure were shown. The RMSD values of the whole structure for WT (blue), T219A (orange), and TPO219 (green), and the partial residues (10–290: without the N- and C-terminal regions) for WT (red), T219A (purple), and TPO219 (brown) are shown. (D) Structural comparison between PP2Ac WT and TPO219. The initial structure for MD simulation, 1-μsec structure of WT and TPO219 are shown in yellow, cyan, and red, respectively. The dotted boxes indicate the regions A, B, and C of the three characteristic peaks. The two $Mn^{2+}$ are shown by ball representation. The inset is an enlarged view of regions B and C. The amino acid residues at 219 are indicated by stick representation. (E) An enlarged view of the modeled complex of the simulation structure of the PP2Ac TPO219 binding to PME-1. R214 formed salt bridges with the phosphorylated Thr219.

Supplemental Figure 6

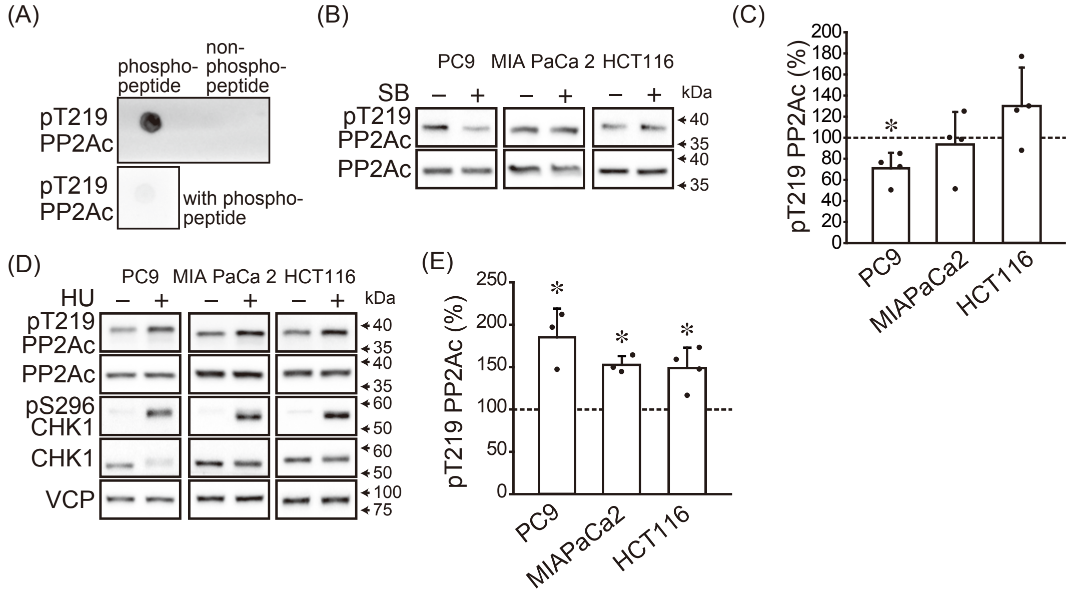


**Supplemental Figure 6**

(A) Dot blotting was performed to confirm the specificity of the anti-phospho-Thr219 PP2Ac antibody. (B–E) Indicated cell lines were treated with SB218078 (5 µM, 8 h) (B–C) and hydroxyurea (HU, 2 mM, 24 h) (D–E). Immunoblotting was performed to analyze PP2Ac phosphorylation levels at Thr219. Representative images (B, D) and quantitative data (C, E) were shown. *: $P < 0.05$. Data points are independent biological replicates.

Supplemental Figure 7

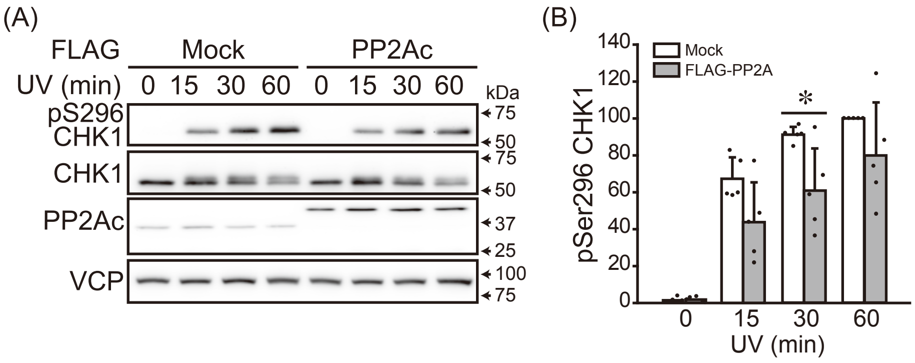


**Supplemental Figure 7**

(A–B) A549 cells stably expressing mock and FLAG-PP2Ac were exposed to UV radiation. Immunoblotting was performed to analyze CHK1 phosphorylation levels. Representative images (A) and quantitative data (B) are shown. *: $P < 0.05$. Data points are independent biological replicates.
